# Interleukin-1 Polymorphisms in Caucasian Adults with Down Syndrome and Advanced Periodontitis: A Cross-Sectional Study

**DOI:** 10.3390/dj14050246

**Published:** 2026-04-22

**Authors:** Marco Montevecchi, Leoluca Valeriani

**Affiliations:** 1 Department of Biomedical and Neuromotor Sciences, Division of Periodontology and Implantology, School of Dentistry, Alma Mater Studiorum, University of Bologna, 40138 Bologna, Italy; leoluca.valeriani2@unibo.it; 2 Department of Theoretical and Applied Sciences, e-Campus University, 22060 Novedrate, Italy

**Keywords:** Down syndrome, periodontitis, interleukin-1 polymorphisms, genetic susceptibility

## Abstract

**Background/Objectives:** Down syndrome (DS) is characterised by a marked susceptibility to early-onset severe periodontitis, suggesting an intrinsic host predisposition. Interleukin-1 (IL-1) gene variants may influence inflammatory burden, yet DS-specific evidence is limited. **Methods:** Nineteen Caucasian adults with DS underwent a comprehensive periodontal examination and received a periodontal diagnosis according to the AAP/EFP 2018 classification. Buccal swabs were genotyped by real-time PCR for *IL1A* −889, *IL1B* +3954 and *IL1RN* +2018; the composite *IL1A*/*B* genotype was also evaluated. **Results:** All participants presented advanced, generalized periodontitis (Stage III/IV: 37%/63%; Grade B/C: 32%/68%). Variant alleles were detected in 63% for *IL1A*, 53% for *IL1B* and 37% for *IL1RN*, and the composite IL-1A/B genotype in 47%. Variant carriage showed associations with higher Clinical Attachment Loss (*IL1A p* = 0.03; *IL1B p* = 0.002; composite *p* = 0.012) and Bleeding on Probing (*IL1A p* = 0.02; *IL1RN p* = 0.05; composite *p* = 0.04). The composite genotype was associated with Stage IV (*p* = 0.027) and Grade C (*p* = 0.005), and tooth loss was greater among variant carriers for all polymorphisms (*p* = 0.01). **Conclusions:** In this DS cohort with advanced periodontitis, IL-1 variants (particularly the composite *IL1A*/*B* genotype) were frequently observed and were associated with greater periodontal severity and tooth loss.

## 1. Introduction

Down syndrome (DS), caused by trisomy of chromosome 21, is the most common human aneuploidy compatible with long-term survival. From an oral-health standpoint, individuals with DS typically present a distinctive profile, characterised by a high susceptibility to early-onset and severe periodontal disease, alongside a comparatively lower risk of dental caries than non-affected peers [1,2,3].

Periodontitis is initiated by a dysbiotic subgingival biofilm, but its severity and rate of progression may be influenced by the magnitude and regulation of the host inflammatory response. In DS, several non-mutually exclusive mechanisms have been proposed to account for the increased susceptibility to periodontal destruction, including impaired or altered immune responses, increased tissue reactivity, and features of premature senescence [2,4].

Microbiological evidence supports that DS subjects may harbour putative periodontal pathogens early in life, even when clinically detectable periodontal breakdown is absent [4,5,6,7,8]. PCR-based studies in primary dentition further support early colonisation in DS: in children without periodontal breakdown, probing depth and bleeding were comparable to controls, yet gingival inflammation was higher, and Aggregatibacter actinomycetemcomitans and Tannerella forsythia were detected more frequently (approximately 8- and 9-fold, respectively) [9].

Microbiome analyses also indicate that DS individuals with periodontitis have a distinct subgingival microbial profile compared with DS individuals without periodontitis [10]. Overall, these findings support the hypothesis that early microbial colonisation may represent one component of an intrinsic predisposition to periodontal disease in DS.

Nevertheless, early exposure to periodontal pathogens alone is unlikely to fully explain the marked heterogeneity in periodontal destruction and tooth loss observed among adults with DS. Inter-individual differences in host susceptibility, partly shaped by genetic factors, are likely to contribute to variation in inflammatory response amplitude and tissue breakdown. In this context, individuals with DS frequently exhibit impaired immune defences, characterized by both quantitative and functional neutrophil defects, including reduced chemotaxis and phagocytic activity [11,12]. In parallel, a systemic and local pro-inflammatory profile has been described in DS, with increased circulating cytokines and, in some reports, higher gingival crevicular fluid inflammatory mediator levels compared with controls, consistent with enhanced periodontal tissue reactivity [13,14].

The mechanisms of inflammation involve numerous pro-inflammatory mediators; hence, an increased risk of periodontitis may be related to the aberrant production of pro-inflammatory cytokines [15].

Among candidate host determinants, polymorphisms within the interleukin-1 (IL-1) gene cluster have been extensively studied in periodontology. IL-1 consists of 11 genes in the 430-kb fragment in the long-arm DNA of chromosome 2 in the 2q12-q21 region [16]. This gene cluster encodes interleukin-1 alpha (IL-1α), interleukin-1 beta (IL-1β), and the interleukin-1 receptor antagonist (IL-1Ra) [17]. IL-1α and IL-1β play a pro-inflammatory role in the pathogenesis of periodontitis, whereas IL-1Ra acts antagonistically to these cytokines [18]. IL-1 participates in a number of processes necessary to initiate and sustain an inflammatory response. It increases the production of adhesion molecules, facilitating leukocyte migration, stimulates the production of other inflammatory mediators and metalloproteinases, actives T and B lymphocytes, stimulates osteoblasts leading to bone resorption, and stimulates the programmed death of cells producing extracellular matrix, thus limiting the regenerative capabilities of tissues.

In periodontal tissues, bacterial exposure elicits a coordinated host immune-inflammatory response in which interleukins play a central regulatory role [19]. These mediators activate signalling pathways that enhance communication between innate and adaptive immune compartments, promoting lymphocyte activation, neutrophil recruitment, and the formation and activity of osteoclasts, multinucleated cells regarded as the major effectors of bone resorption [20,21].

Although the inflammatory response is intended to contain the microbial challenge that initiates and perpetuates the disease, failure of its resolution may lead to chronic inflammation [22]. In this condition, the response may become dysregulated, and the fragile balance between tissue destruction and healing may shift toward further destruction [23]. IL-1 α is a key mediator of the host immune response, constitutively expressed under healthy conditions and further upregulated during inflammation, where it enhances cytokine release and neutrophil recruitment. IL-1 β shares several biological functions with IL-1 α and binds to the same receptor, although it acts through distinct pathophysiological mechanisms.

Specific variants, commonly investigated at *IL1A* −889, *IL1B* +3954, and *IL1RN* +2018, have been associated in various clinical investigations [24,25,26,27] with a more severe periodontal phenotype, particularly in Caucasian populations; additionally, a “composite” IL-1 genotype (co-occurrence of variant alleles at *IL1A* −889 and *IL1B* +3954) has been proposed as a severity factor in adult periodontal disease [24,25].

Despite the biological plausibility of an IL-1-mediated susceptibility contribution in DS, evidence in DS remains limited and not fully consistent. To date, only scarce DS-focused data have addressed IL-1 polymorphisms in relation to periodontal outcomes [28].

Moreover, the availability of contemporary periodontal diagnostic frameworks based on staging and grading provides an opportunity to examine genetic associations using clinically meaningful disease stratification [29].

Therefore, the aim of this study is to explore, through genetic analysis, the distribution of specific interleukin-1 polymorphisms in adult individuals with Down syndrome and periodontitis.

## 2. Material and Methods

### 2.1. Study Sample

Patients were enrolled using a consecutive convenience sampling approach from attendees of the Dentistry Service for Individuals with Disabilities, Dental Clinic, Department of Biomedical and Neuromotor Sciences, University of Bologna, Italy, who presented for initial visit, routine dental care or other clinical needs between 3 September 2018 and 16 September 2019. This timeframe corresponded to the active recruitment phase of the research project, and all eligible patients available during this window were included to maximize the study sample. Eligibility was assessed according to the following inclusion and exclusion criteria.

Inclusion criteria were:

- Chromosomally confirmed diagnosis of Down syndrome;
- Diagnosis of periodontitis according to the 2018 AAP/EFP case definition [29];
- Caucasian adults (≥18 years);
- Availability of a recent panoramic radiograph (orthopantomogram) of the dental arches, performed within the previous 6 months;
- Presence of at least two pairs of teeth;
- Informed consent obtained prior to enrollment.

Exclusion criteria were:

- Current smoking or history of smoking;
- Current use of medications with a clear influence on periodontal status;
- Presence of systemic diseases and/or conditions with a clear influence on periodontal status (e.g., diabetes, acquired immunodeficiencies, hematological disorders, and conditions affecting immune response).

Specific informed consent was obtained from the legal guardian of each participant enrolled in the study; participants also received an understandable explanation of the study and provided their agreement to participate, and any refusal to participate was respected. The Ethics Committee for Research of Sant’Orsola-Malpighi Hospital, Bologna, Italy, approved the study (PG. N. 0019293, 20 June 2014).

### 2.2. Clinical Examination

An experienced periodontist (M.M.) performed a comprehensive periodontal screening for each participant enrolled in the study. After dental charting, the number and the reasons for the loss of any missing teeth were investigated. Third molars were not included in the analysis.

Using a periodontal probe (CP-12 UNC SE, Hu-Friedy, Chicago, IL, USA), probing pocket depth and gingival recession were recorded at six sites for each tooth present. The presence and extent of furcation involvement were assessed using a calibrated periodontal probe (PQ2N6 Nabers probe, Hu-Friedy, Chicago, IL, USA). Clinical attachment level (CAL) was calculated, and bleeding on probing (BOP) [30], plaque index (PI) [31], and tooth mobility [32] were recorded. A periodontitis case was defined by the presence of interdental CAL at ≥2 non-adjacent teeth or buccal/oral CAL ≥3 mm with pocketing ≥3 mm at ≥2 teeth, not attributable to non-periodontitis-related causes [29]. Following completion of the medical history assessment and clinical examination, periodontal stage and grade were determined according to the 2018 Classification of Periodontal and Peri-Implant Diseases and Conditions [33].

### 2.3. Genetic Analysis

The sample for genetic analysis was collected by the same periodontist during the periodontal screening, after clinical data collection.

Buccal mucosal epithelial cells were obtained by brushing with a sterile swab (Copan Group, Brescia, Italy). The swab was then placed in a dedicated transport container and sent to an external laboratory (Biomolecular Diagnostic, Florence, Italy) for subsequent analyses.

Upon arrival at the laboratory, DNA was extracted using the QIAcube HT^®^ instrument (Qiagen, Hilden, Germany), strictly following the manufacturer’s protocol. After extraction, DNA was eluted in 150 µL of buffer and DNA concentration was measured (ng/µL) using a BioSpectrometer^®^ (Eppendorf, Hamburg, Germany). An amount corresponding to 40 ng of DNA was used to analyze the following IL-1 genetic polymorphisms (*IL1A* −889, *IL1B* +3954, and *IL1RN* +2018) by real-time PCR. The *IL1A* −889, *IL1B* +3954, and *IL1RN* +2018 polymorphisms were selected due to their functional relevance within the IL-1 pathway, influencing cytokine production and regulation, and because they represent the most extensively investigated loci in the IL-1 gene cluster, with consistent evidence supporting their association with periodontal disease susceptibility [25,27,34,35].

The probe sequences used (Life Technologies, Carlsbad, CA, USA) are reported in Table 1. Genetic analyses were performed blinded to the clinical data of the sample, and the periodontal examination and clinical staging were completed before the genetic results became available.

### 2.4. Statistical Analysis

Frequency distributions and contingency tables, as well as arithmetic means with standard deviations or medians with interquartile ranges (according to data distribution), were used to describe qualitative and quantitative variables. Depending on data distribution, comparisons were performed using Student’s *t* test or the Mann–Whitney U test; the chi-square test was used to assess associations in contingency tables. The alpha level was set a priori at 0.05. All analyses were perfomed using IBM SPSS Statistics 31.0.0 (SPSS Inc., Chicago, IL, USA)

## 3. Results

A total of 19 adults with a chromosomally confirmed diagnosis of Down syndrome were enrolled, including 7 males and 12 females, aged 30–58 years (mean age: 46 ± 7 years). Mean CAL was 4.77 (±0.96 mm), mean PI was 49% (±15), and mean BOP was 43% (±11).

Notably, no participants met the criteria for Stage I or II periodontitis. Seven participants (37%) were classified as Stage III and 12 (63%) as Stage IV, and all presented with generalized periodontitis (extent ≥30% of teeth present). Grade assignment identified 6 (32%) participants as grade B and 13 (68%) as grade C.

The median number of teeth present was 24 [22.5–27]. All tooth loss was attributed to periodontal reasons. Two patients exhibited dental agenesis, accounting for a total of three missing teeth. The median number of teeth lost was 3 [1,2,3,4,5,6].

The minor *IL1A* polymorphism was detected in 12 participants (63%), the minor *IL1B* polymorphism in 10 (53%), and the minor *IL1RN* polymorphism in 7 (37%). The composite condition of the minor *IL1A/B* allele was observed in 9 participants (47%).

The associations between IL-1 polymorphisms and the periodontal variables investigated are reported in Table 2.

## 4. Discussion

For the development of periodontitis, the current literature indicates that disease progression is driven by a dysbiotic microbial community rather than any single specific pathogen, while host genetic and environmental factors also significantly influence disease severity [29,36]. Consistently, the relevance of genetic predisposition has been clearly demonstrated by investigations in monozygotic individuals, suggesting that approximately 50% of the variability in the clinical phenotype of periodontitis may be genetically determined [37].

From a genetic perspective, one of the most extensively studied cytokines in periodontal research is interleukin-1, which comprises at least 10 different molecules. Among these, those currently considered most relevant to periodontal disease include IL-1α (*IL1A* gene), IL-1β (*IL1B* gene), and the related interleukin-1 receptor antagonist (IL-1Ra), which is encoded by the *IL1RN* gene [24,25,27].

IL-1 is a key regulator of the host inflammatory response and plays a central role in the pathogenesis of periodontal disease. IL-1α and IL-1β are produced by activated macrophages, monocytes, dendritic cells, neutrophils, and resident cells such as gingival fibroblasts and epithelial cells in response to periodontal pathogens [38,39]. These cytokines promote leukocyte recruitment, upregulate adhesion molecules, and stimulate the production of matrix metalloproteinases and prostaglandins, thereby contributing to connective tissue breakdown [38]. Moreover, IL-1β enhances RANKL expression and osteoclast differentiation, ultimately leading to alveolar bone resorption [39,40].

The association between IL-1 genetic variants and chronic periodontitis in Caucasian individuals was first reported in 1997 by Kornman et al. [25] in 133 adults aged >35 years of Northern European Caucasian ancestry, including 49 subjects with mild, 42 with moderate, and 42 with severe chronic periodontitis. The authors described a specific IL-1 genetic profile, termed the composite genotype, characterized by the concomitant presence of two polymorphisms, *IL1A* −889 and *IL1B* +3953. In non-smoking subjects aged 40–60 years with severe chronic periodontitis, composite genotype positivity was detected in 78% of cases, corresponding to an approximately sevenfold higher risk compared with controls.

This composite genotype was investigated in the present study and was observed in 47% of the overall sample. When considering participants with stage IV, grade C periodontitis (i.e., the most severe and advanced condition) positivity was detected in 89% (8 of 9). This finding is consistent with the results reported by Kornman et al. [25]. Subsequently, numerous studies have explored the role of IL-1 genetic polymorphisms in periodontitis; however, findings have not always been consistent.

Recent primary studies still support a potential role of IL-1 pathway genetic variability in the etiopathogenesis and clinical expression of periodontitis, with the “IL-1 positive” composite profile (*IL1A* −889 and *IL1B* +3953/+3954) being reported more frequently among periodontitis cases and in advanced disease phenotypes in some cohorts [41,42]. At the same time, population-dependent effects have been described, with a case–control study in an Iraqi cohort reporting a significantly lower frequency of the rs1143634 TT genotype among periodontitis cases (suggesting a protective effect of the T allele), highlighting that the direction and magnitude of association may differ across ethnic backgrounds and study settings [43]. Beyond susceptibility, clinical data also suggest that IL-1 composite genotype status may also influence the early healing dynamics after non-surgical periodontal therapy (although differences appear modest and not always clinically significant) supporting a possible modulatory effect on treatment response [44]. In contrast, evidence for *IL1RN* variants remains less consistent, with some datasets failing to show robust associations compared with *IL1A*/*IL1B* and composite profiles [45].

As highlighted by a review published in 2012 [24], several factors may account for such variability. One major factor is ethnicity, as the distribution of IL-1 polymorphisms differs substantially across populations, necessitating rigorous control of this variable both in study design and when comparing results. In particular, Caucasian individuals show a higher frequency of these polymorphisms than other populations, such as Asian groups. For this reason, the present study was conducted exclusively in Caucasian participants.

Another factor that may influence the results is cigarette smoking, as smoking is recognized as one of the major risk factors for periodontitis [46,47]. Several studies investigating IL-1 polymorphisms and periodontitis have shown that smoking may act as a confounder when assessing the effect of genetic predisposition related to IL-1 variants [25,48]. For this reason, smokers were excluded from the present study.

A further relevant issue concerns the classification criteria historically used for periodontal disease. One of the main reasons that led the 2018 World Workshop in Chicago to reconsider the existing framework and develop a new classification was the difficulty in distinguishing aggressive from chronic forms [33]. Since these two entities were introduced in 1999 [49], insufficient scientific evidence has accumulated to support their interpretation as distinct diseases; therefore, they are currently considered different manifestations of the same condition, influenced by variables that are not yet fully understood. This issue may also explain the lack of association between IL-1 polymorphisms and aggressive periodontitis reported in some studies [50,51]. It has been hypothesized that, in this clinical phenotype, factors with a stronger effect than IL-1 genetic variation may predominate, thereby masking its potential contribution. To date, only one study [52] investigating the association between periodontitis and IL-1 has applied the new classification system. As noted above, this aspect may substantially affect results and the resulting body of evidence, and it also limits direct comparison between the present findings and those available in the literature.

An early study investigating IL-1 polymorphisms in DS was published in 2011 by Khocht et al. [28]. The authors examined the distribution of IL-1 genotypes in individuals with DS and assessed the association between genetic polymorphisms at the *IL1A* +4845 locus (overlapping with *IL1A* −889), *IL1B* +3954, and *IL1RN* +2018 and the degree of periodontal destruction. The sample comprised 54 individuals with DS and was compared with two additional samples matched for sex, age, and ethnicity: 71 individuals with intellectual disability and 87 healthy controls. The DS group included participants of different ethnic backgrounds, with a predominance of Caucasian individuals but without adequate reporting of the remaining ethnicities. When comparing the descriptive characteristics of the DS participants in Khocht et al. [28] with those of the present study, both mean age (35.96 ± 1.64 vs. 46 ± 7 years) and clinical attachment loss (2.66 ± 0.10 vs. 4.77 ± 0.96) were lower in the former. These differences may be explained by the inclusion, unlike the present study, of DS participants without periodontitis (28%). In contrast, the number of missing teeth was higher in Khocht et al. [28] than in the present study (4.57 ± 0.53 vs. 3 [1,2,3,4,5,6]). Because the reasons for tooth absence were not reported, missing teeth due to agenesis or causes other than periodontitis may also have been included. A comparison of other variables assessed in the present study was not feasible due to methodological differences.

Khocht et al. [28] did not report any significant differences among the three groups in the distribution of individual IL-1 polymorphisms or of the composite genotype; however, these findings were presented without numerical data. Compared with the pooled data reported by Khocht et al. [28] across all three samples, the present study showed higher detection rates for *IL1A* (63% vs. 46.8%), *IL1B* (53% vs. 43%), and the composite genotype (47% vs. 32%). A comparison for *IL1RN* was not possible because the corresponding value was not reported.

The higher frequency of IL-1 variant genotypes in our cohort should be interpreted cautiously, as our sample was restricted to Caucasian adults with DS and advanced periodontitis, whereas Khocht et al. [28] included participants with heterogeneous ethnicity and a proportion without periodontitis, which may have diluted associations. In their within-DS analyses, Khocht et al. [28] reported lower gingival inflammation among carriers of the *IL1B* +3954 variant and lower clinical attachment loss among individuals with the composite genotype; they also described an association between *IL1RN* status and attachment loss, overall interpreting IL-1 variants as potentially protective in DS. This interpretation contrasts with our findings, where variant alleles, particularly the composite genotype, were associated with greater periodontal severity and tooth loss, in line with evidence from non-DS populations [24].

Our findings are also in line with recent evidence indicating that DS individuals with periodontitis exhibit marked IL-1β overexpression at the local periodontal level. Theodoro et al. (2026) [52] reported IL-1β concentrations in gingival crevicular fluid that were approximately fivefold higher than those observed in non-DS periodontitis patients and nearly 60-fold higher than those found in healthy controls.

Transcriptomic data provide complementary functional support for the involvement of IL-1-related pathways in high-risk DS phenotypes. In a retrospective case–control study, Baus-Domínguez et al. [53] reported differential expression of *IL1B* and *IL1RN*, together with bone-related genes (BGLAP, PTK2, FOXO1A), when comparing DS individuals with periodontal disease and implant failure to DS controls with favourable implant outcomes. Although gene expression findings cannot be directly extrapolated to germline polymorphisms, these results may strengthen the biological plausibility of IL-1 axis dysregulation in severe DS periodontal/peri-implant presentations and support the rationale for integrative genotype–phenotype investigations.

Although this study provides data on specific genetic variants in a DS population, some limitations must be acknowledged. First and foremost, the sample size was relatively small, which may affect the generalizability of the results and the ability to identify subtle genetic associations. However, it should be noted that DS is a relatively rare condition, and recruiting a highly homogeneous cohort (e.g., Caucasian, non-smokers, specific age range) presents significant logistical and clinical challenges.

Furthermore, the cross-sectional nature of this study prevents us from establishing a definitive causal relationship between the identified variants and the rate of periodontal progression. Additionally, although we controlled for major confounders like smoking and systemic health, other environmental or lifestyle factors (such as specific oral hygiene habits or professional plaque control frequency) were not fully accounted for and could influence the clinical phenotype.

Given that genetic research typically requires large study samples [54], the present findings should be interpreted as exploratory and hypothesis-generating. Multicentre studies are warranted to increase the sample size, together with longitudinal investigations to better define the role of these variants in the periodontal disease course in this specific population.

## 5. Conclusions

Specific polymorphisms of IL-1α, IL-1β, and IL-1Ra, which have been associated with periodontitis in the general population, were observed in this Caucasian adult DS cohort with severe periodontal destruction (stage III/IV, grade B/C) in a substantial proportion of participants. These variants, and especially the composite genotype, were also exploratorily associated with the severity of the clinical presentation within this sample.

Overall, these findings suggest that IL-1-related genetic variation may be linked to periodontal severity in DS; however, the present data do not allow inferences on causality or on genetic predisposition at the population level. Longitudinal, multicenter studies are required to better clarify the role and potential predictive value of these etiopathogenetic variables in the progression of periodontal disease.

Based on current evidence, close periodontal monitoring combined with rigorous oral hygiene control from early childhood is advisable, in line with established preventive care for individuals with DS and their recognised periodontal vulnerability.

## Figures and Tables

**Table 1:** Probe sequences used for real-time PCR genotyping of IL-1 polymorphisms.

Target	Sequence (5′-3′)	Labelling
*IL1A*	GATTTTTACATATGAGCCTTCAATG[G/A]TGTTGCCTGGTTACTATTATTAAAG	VIC/FAM
*IL1B*	CATAAGCCTCGTTATCCCATGTGTC[G/A]AAGAAGATAGGTTCTGAAATGTGGA	VIC/FAM
*IL1RN*	ATCTGAGGAACAACCAACTAGTTGC[C/T]GGATACTTGCAAGGACCAAATGTCA	VIC/FAM

[G/A] = nucleotide substitution G/A (polymorphic site). [C/T] = nucleotide substitution C/T (polymorphic site).

**Table 2:** Associations between IL-1 polymorphisms and indicators of periodontal severity and inflammatory burden.

	*IL1A*			*IL1B*			*IL1RN*			*IL1A/B*		
	Common	Variant	*p*-Value	Common	Variant	*p*-Value	Common	Variant	*p*-Value	Common	Variant	*p*-Value
CAL mean (SD)	4.21 (0.81)	5.19 (0.91)	0.03	4.15 (0.66)	5.37 (0.77)	0.002	4.7 (0.86)	4.89 (1.17)	NS	4.27 (0.82)	5.32 (0.80)	0.012
BoP mean (SD)	35 (10.19)	48.16 (9.57)	0.02	39 (10.25)	47.4 (11.50)	NS	39 (8.58)	51 (12.37)	0.05	38 (9.91)	49 (10.76)	0.04
Stage n	Stage III		NS	Stage III		NS	Stage III		NS	Stage III		0.027
	4	3		5	2		5	2		1	6	
	Stage IV			Stage IV			Stage IV			Stage IV		
	3	9		4	8		7	5		8	4	
Grade n	Grade B		0.0001	Grade B		0.002	Grade B		NS	Grade B		0.005
	6	0		6	0		5	1		0	6	
	Grade C			Grade C			Grade C			Grade C		
	1	12		3	19		7	6		9	4	
Tooth loss * median (IQR)	1(1)	6(4)	0.0001	1(1)	7(4)	0.001	3(3)	8(4)	0.01	1(1)	7(4)	0.01

Abbreviations/notes: CAL: clinical attachment level, mm; BOP: bleeding on probing, %; NS: not statistically significant (*p* > 0.05); SD: standard deviation; IQR: Interquartile range; * Mann–Whitney U test.

## Data Availability

The data presented in this study are available on request from the corresponding author. Data are not publicly available due to privacy or ethical restrictions.

## References

[1] de Castilho A.R.F., Marta S.N. (2010). Evaluation of the incidence of dental caries in patients with Down syndrome after their inser-tion in a preventive program. Ciência Saúde Coletiva.

[2] Jepsen S., Caton J.G., Albandar J.M., Bissada N.F., Bouchard P., Cortellini P., Demirel K., de Sanctis M., Ercoli C., Fan J. (2018). Periodontal manifestations of systemic diseases and developmental and acquired conditions: Consensus report of workgroup 3 of the 2017 World Workshop on the Classification of Periodontal and Peri-Implant Diseases and Conditions. J. Clin. Periodontol..

[3] Nuernberg M.A.A., Ivanaga C.A., Haas A.N., Aranega A.M., Casarin R.C.V., Caminaga R.M.S., Garcia V.G., Theodoro L.H. (2019). Periodontal status of individuals with Down syndrome: Sociodemographic, behavioural and family perception influence. J. Intellect. Disabil. Res..

[4] Amano A., Kishima T., Akiyama S., Nakagawa I., Hamada S., Morisaki I. (2001). Relationship of periodontopathic bacteria with early-onset periodontitis in Down’s syndrome. J. Periodontol..

[5] Amano A., Kishima T., Kimura S., Takiguchi M., Ooshima T., Hamada S., Morisaki I. (2000). Periodontopathic bacteria in children with Down syndrome. J. Periodontol..

[6] Morinushi T., Lopatin D.E., Van Poperin N. (1997). The Relationship Between Gingivitis and the Serum Antibodies to the Microbiota Associated With Periodontal Disease in Children With Down’s Syndrome. J. Periodontol..

[7] Naka S., Yamana A., Nakano K., Okawa R., Fujita K., Kojima A., Nemoto H., Nomura R., Matsumoto M., Ooshima T. (2009). Distribution of periodontopathic bacterial species in Japanese children with developmental disabilities. BMC Oral Health.

[8] Sakellari D., Belibasakis G., Chadjipadelis T., Arapostathis K., Konstantinidis A. (2001). Supragingival and subgingival microbiota of adult patients with Down’s syndrome. Changes after periodontal treatment. Oral Microbiol. Immunol..

[9] Vocale C., Montevecchi M., D’Alessandro G., Gatto M., Piana G., Nibali L., Re M.C., Sambri V. (2021). Subgingival periodontal pathogens in Down syndrome children without periodontal breakdown. A case-control study on deciduous teeth. Eur. J. Paediatr. Dent..

[10] Nóvoa L., Sánchez M.D.C., Blanco J., Limeres J., Cuenca M., Marín M.J., Sanz M., Herrera D., Diz P. (2020). The Subgingival Microbiome in Patients with Down Syndrome and Periodontitis. J. Clin. Med..

[11] Ramba M., Bogunovic D. (2024). The immune system in Down Syndrome: Autoimmunity and severe infections. Immunol. Rev..

[12] Ram G., Chinen J. (2011). Infections and immunodeficiency in Down syndrome. Clin. Exp. Immunol..

[13] Tsilingaridis G., Yucel-Lindberg T., Modéer T. (2012). T-helper-related cytokines in gingival crevicular fluid from adolescents with Down syndrome. Clin. Oral Investig..

[14] Zhang Y., Che M., Yuan J., Yu Y., Cao C., Qin X.Y., Cheng Y. (2017). Aberrations in circulating inflammatory cytokine levels in patients with Down syndrome: A meta-analysis. Oncotarget.

[15] Page R.C., Offenbacher S., Schroeder H.E., Seymour G.J., Kornman K.S. (1997). Advances in the pathogenesis of periodontitis: Summary of developments, clinical implications and future directions. Periodontol. 2000.

[16] Nicklin M.J., Weith A., Duff G.W. (1994). A physical map of the region encompassing the human interleukin-1 alpha, interleukin-1 beta, and interleukin-1 receptor antagonist genes. Genomics.

[17] Deng J.S., Qin P., Li X.X., Du Y.H. (2013). Association between interleukin-1β C (3953/4)T polymorphism and chronic periodontitis: Evidence from a meta-analysis. Hum. Immunol..

[18] Rawlinson A., Grummitt J.M., Walsh T.F., Ian Douglas C.W. (2003). Interleukin 1 and receptor antagonist levels in gingival crevicular fluid in heavy smokers versus non-smokers. J. Clin. Periodontol..

[19] Nędzi-Góra M., Kowalski J., Górska R. (2017). The Immune Response in Periodontal Tissues. Arch. Immunol. Ther. Exp..

[20] Bartold P.M., Cantley M.D., Haynes D.R. (2010). Mechanisms and control of pathologic bone loss in periodontitis. Periodontol. 2000.

[21] Tanabe N., Maeno M., Suzuki N., Fujisaki K., Tanaka H., Ogiso B., Ito K. (2005). IL-1 alpha stimulates the formation of osteoclast-like cells by increasing M-CSF and PGE2 production and decreasing OPG production by osteoblasts. Life Sci..

[22] Yucel-Lindberg T., Båge T. (2013). Inflammatory mediators in the pathogenesis of periodontitis. Expert Rev. Mol. Med..

[23] Pan W., Wang Q., Chen Q. (2019). The cytokine network involved in the host immune response to periodontitis. Int. J. Oral Sci..

[24] Karimbux N.Y., Saraiya V.M., Elangovan S., Allareddy V., Kinnunen T., Kornman K.S., Duff G.W. (2012). Interleukin-1 gene polymor-phisms and chronic periodontitis in adult whites: A systematic review and meta-analysis. J. Periodontol..

[25] Kornman K.S., Crane A., Wang H.Y., di Giovine F.S., Newman M.G., Pirk F.W., Wilson T.G., Higginbottom F.L., Duff G.W. (1997). The in-terleukin-1 genotype as a severity factor in adult periodontal disease. J. Clin. Periodontol..

[26] López N.J., Jara L., Valenzuela C.Y. (2005). Association of interleukin-1 polymorphisms with periodontal disease. J. Periodontol..

[27] da Silva F.R.P., dos Santos Pessoa L., Shin J.I., Alves E.H.P., Koga R.S., Smith C.V., Vasconcelos D.F.P., da Cunha Pereira A.C.T. (2021). Polymorphisms in the interleukin genes and chronic periodontitis: A field synopsis and revaluation by Bayesian approaches. Cytokine.

[28] Khocht A., Heaney K., Janal M., Turner B. (2011). Association of interleukin-1 polymorphisms with periodontitis in Down syn-drome. J. Oral Sci..

[29] Papapanou P.N., Sanz M., Buduneli N., Dietrich T., Feres M., Fine D.H., Flemmig T.F., Garcia R., Giannobile W.V., Graziani F. (2018). Periodontitis: Consensus report of workgroup 2 of the 2017 World Workshop on the Classification of Periodontal and Peri-Implant Diseases and Conditions. J. Clin. Periodontol..

[30] Guerrero A., Griffiths G.S., Nibali L., Suvan J., Moles D.R., Laurell L., Tonetti M.S. (2005). Adjunctive benefits of systemic amoxicillin and metronidazole in non-surgical treatment of generalized aggressive periodontitis: A randomized placebo-controlled clinical trial. J. Clin. Periodontol..

[31] O’Leary T.J., Drake R.B., Naylor J.E. (1972). The plaque control record. J. Periodontol..

[32] Miller S.C. (1938). Textbook of Periodontia (Oral Medicine).

[33] Tonetti M.S., Greenwell H., Kornman K.S. (2018). Staging and grading of periodontitis: Framework and proposal of a new classification and case definition. J. Clin. Periodontol..

[34] McDevitt M.J., Wang H.Y., Knobelman C., Newman M.G., di Giovine F.S., Timms J., Duff G.W., Kornman K.S. (2000). Interleukin-1 genetic association with periodontitis in clinical practice. J. Periodontol..

[35] Diehl S.R., Wang Y., Brooks C.N., Burmeister J.A., Califano J.V., Wang S., Schenkein H.A. (1999). Linkage disequilibrium of interleukin-1 genetic polymorphisms with early-onset periodontitis. J. Periodontol..

[36] Hajishengallis G. (2014). Immunomicrobial pathogenesis of periodontitis: Keystones, pathobionts, and host response. Trends Immunol..

[37] Michalowicz B.S., Diehl S.R., Gunsolley J.C., Sparks B.S., Brooks C.N., Koertge T.E., Califano J.V., Burmeister J.A., Schenkein H.A. (2000). Evi-dence of a substantial genetic basis for risk of adult periodontitis. J. Periodontol..

[38] Graves D.T., Cochran D. (2003). The contribution of interleukin-1 and tumor necrosis factor to periodontal tissue destruction. J. Periodontol..

[39] Dinarello C.A. (2018). Overview of the IL-1 family in innate inflammation and acquired immunity. Immunol. Rev..

[40] Shapira L., Wilensky A., Kinane D.F. (2021). Effect of genetic variability on the inflammatory response to periodontal infection. Periodontol. 2000.

[41] Brodzikowska A., Górski B., Bogusławska-Kapała A. (2022). Association between IL-1 Gene Polymorphisms and Stage III Grade B Periodontitis in Polish Population. Int. J. Environ. Res. Public Health.

[42] Majeed M.M., Ahmed I., Roome T., Fatima T., Amin R. (2021). Association between Interleukin-1β Gene Polymorphism and Chronic Periodontitis. Eur. J. Dent..

[43] Dahash S.A., Kusrat Hussein L. (2023). Interleukin-1β rs1143634 Polymorphism and Susceptibility to Periodontitis in the Iraqi Pop-ulation. Arch. Razi Inst..

[44] Brodzikowska A., Górski B., Bogusławska-Kapała A. (2023). Effects of Interleukin-1 Genotype on the Clinical Efficacy of Non-Surgical Periodontal Treatment of Polish Patients with Periodontitis. Biomed.

[45] Albu Ş-D., Dragomirescu A.-O., Albu C.-C., Ionescu E. (2024). Genetic Polymorphisms of Interleukins IL-1a, IL-1b, and IL-1rn in Patients with Periodontal Disease and Dento-Maxillary Anomalies. Rom. J. Oral Rehabil..

[46] Haber J. (1994). Smoking is a major risk factor for periodontitis. Curr. Opin. Periodontol..

[47] Kinane D.F., Chestnutt I.G. (2000). Smoking and periodontal disease. Crit. Rev. Oral Biol. Med..

[48] Pretzl B., El Sayed N., Cosgarea R., Kaltschmitt J., Kim T.S., Eickholz P., Nickles K., Bäumer A. (2012). IL-1-polymorphism and severity of periodontal disease. Acta Odontol. Scand..

[49] Armitage G.C. (1999). Development of a classification system for periodontal diseases and conditions. Ann. Periodontol..

[50] Fiebig A., Jepsen S., Loos B.G., Scholz C., Schäfer C., Rühling A., Nothnagel M., Eickholz P., van der Velden U., Schenck K. (2008). Polymorphisms in the interleukin-1 (IL1) gene cluster are not associated with aggressive periodon-titis in a large Caucasian population. Genomics.

[51] Scapoli C., Borzani I., Guarnelli M.E., Mamolini E., Annunziata M., Guida L., Trombelli L. (2010). IL-1 gene cluster is not linked to aggressive periodontitis. J. Dent. Res..

[52] Theodoro L.H., Rodrigues J.V.S., Nuernberg M.A.A., Petrilli P.H., Monteiro M.F., Garcia V.G., de Molon R.S., Casarin R.C.V. (2026). Surveying immune and inflammatory alterations in periodontitis among individuals with Down syndrome: A preliminary cross-sectional study. J. Periodontol..

[53] Baus-Domínguez M., Gómez-Díaz R., Torres-Lagares D., Gutiérrez-Pérez J.-L., Machuca-Portillo G., Serrera-Figallo M.-Á. (2023). Retrospective Case-Control Study Genes Related to Bone Metabolism That Justify the Condition of Periodontal Disease and Failure of Dental Implants in Patients with Down Syndrome. Int. J. Mol. Sci..

[54] Ioannidis J.P.A., Trikalinos T.A., Ntzani E.E., Contopoulos-Ioannidis D.G. (2003). Genetic associations in large versus small stud-ies: An empirical assessment. Lancet.

